# Longitudinal Projection of Herd Prevalence of Influenza A(H1N1)pdm09 Virus Infection in the Norwegian Pig Population by Discrete-Time Markov Chain Modelling

**DOI:** 10.3390/idr13030070

**Published:** 2021-08-25

**Authors:** Jwee Chiek Er

**Affiliations:** Department of Epidemiology, Norwegian Veterinary Institute, Postboks 64, 1433 Ås, Norway; chiek.er@vetinst.no

**Keywords:** discrete-time Markov chain, influenza A(H1N1)pdm09, active serosurveillance, pigs, probability transition matrix

## Abstract

In order to quantify projections of disease burden and to prioritise disease control strategies in the animal population, good mathematical modelling of infectious disease dynamics is required. This article investigates the suitability of discrete-time Markov chain (DTMC) as one such model for forecasting disease burden in the Norwegian pig population after the incursion of influenza A(H1N1)pdm09 virus (H1N1pdm09) in Norwegian pigs in 2009. By the year-end, Norway’s active surveillance further detected 20 positive herds from 54 random pig herds, giving an estimated initial population prevalence of 37% (95% CI 25–52). Since then, Norway’s yearly surveillance of pig herd prevalence has given this study 11 years of data from 2009 to 2020 to work with. Longitudinally, the pig herd prevalence for H1N1pdm09 rose sharply to >40% in three years and then fluctuated narrowly between 48% and 49% for 6 years before declining. This initial longitudinal pattern in herd prevalence from 2009 to 2016 inspired this study to of test the steady-state discrete-time Markov chain model in forecasting disease prevalence. With the pig herd as the unit of analysis, the parameters for DTMC came from the initial two years of surveillance data after the outbreak, namely vector prevalence, first herd incidence and recovery rates. The latter two probabilities formed the fixed probability transition matrix for use in a discrete-time Markov chain (DTMC) that is quite similar to another compartmental model, the susceptible–infected–susceptible (SIS) model. These DTMC of predicted prevalence (DTMCP) showed good congruence (Pearson correlatio*n* = 0.88) with the subsequently observed herd prevalence for seven years from 2010 to 2016. While the DTMCP converged to the stationary (endemic) state of 48% in 2012, after three time steps, the observed prevalence declined instead from 48% after 2016 to 25% in 2018 before rising to 29% in 2020. A sudden plunge in H1N1pdm09 prevalence amongst Norwegians during the 2016/2017 human flu season may have had a knock-on effect in reducing the force of infection in pig herds in Norway. This paper endeavours to present the discrete-time Markov chain (DTMC) as a feasible but limited tool in forecasting the sequence of a predicted infectious disease’s prevalence after it’s incursion as an exotic disease.

## 1. Introduction

Modelling infectious disease dynamics in endemic diseases at the population level is useful and necessary in predicting the disease burden longitudinally caused by the sum total of negative effects of animal diseases. With compartmental models, such as the SIR (susceptible–infected–recovered), SIS (susceptible–infected–susceptible) or Markov chain (MC) models, one can predict disease spread, incidence, prevalence and the duration of the epidemic [1,2]. Forecasting such quantitative information using the right predictive models can help animal health authorities formulate and prioritise control strategies. Fundamental to two closely related models, the MC and SIS, are probabilistic parameters—infection rates and recovery rates, where the element of time determines the two interchangeable disease states (infected or uninfected) for the pig herd. Accurate predictions from these models are dependent on correct assumptions and correct disease dynamics parameters. In the MC model, the two key parameters are the initial vector prevalence and the fixed probability transition matrix, derived from early active serosurveillance data.

Started in 1997, Norway’s active serosurveillance detected the first influenza A virus (IAV) infection in pigs on 30 September 2009 [3,4], at a time when H1N1pdm09 had already spread globally in the human population since emergence in April 2009 because of efficient human-to-human transmission and air travel [5,6,7,8]. The World Health Organisation (WHO) on 11 June 2009 declared H1N1pdm09 a new human pandemic influenza and eventually assigned it the highest level in pandemic classification [9,10,11,12]. By the end of December 2009, over 200 countries were reporting human infections [13].

In Norway, the virus first circulated in the human population in May 2009 [14], four months before the outbreak occurred in the pig population. Targeted and continued active serosurveillance soon unveiled more infected pig herds even though infected pigs had only mild to undetected clinical signs [15,16]. By end of 2009, one-third (*n* = 20) of 54 randomly selected pig herds across Norway tested positive for presence of antibodies [15]. The national prevalence (Table 1) rose quickly in two years to more than 40% by 2011 [17].

The chronological principle of cause-and-effect in epidemiology suggested that humans were the source of the initial infections in pigs. A case–control study supported this reverse zoonosis hypothesis by showing that a history of sick farm workers showing influenza-like symptoms was the most important risk factor (OR > 4) for Norwegian pig herds [18]. A third evidence of reverse zoonosis was a match in genetic sequencing of the H1N1pdm09 viruses extracted from the human and pig hosts [4].

Although influenza in pigs is a list-B disease in Norway, the Norwegian Food Safety Authority (FSA) after slaughtering out one positive pig farm, abandoned further eradication efforts after discovering more infected herds had already spread across Norway in a short space of time making the scope and logistics of eradication cost prohibitive [15]. With the virus already widespread in the human population, unilateral control strategies in pigs could be futile. As preventive measures, FSA advised farmers to avoid contact with pigs if they were sick with influenza-like symptoms and diligently maintain their annual influenza vaccinations [18]. Since infected pigs were largely subclinical and the infection caused little or no mortality, the FSA opted for a wait-and-see approach and was hopeful that the infection would resolve by itself and disappear in time [15,16].

Despite the mild or undetectable clinical signs in infected pigs, a subsequent large longitudinal growth study involving 1955 fattening pigs at a boar testing station spanning four years proved that H1N1pdm09 infection in growing pigs can cause poorer growth rates and protracted growth periods because of lower feed efficiency [19]. When compared to healthy pigs, stochastic models predicted an infected batch of 150 fattening pigs in Norway could consume additional feed ranging from 0.8 to 1.4 (5th–95th percentile) tonnes to reach the market weight of 100 kg per pig [20]. The extra feed represents four to six percent above the normal average ~22 tonnes of feed requirement to grow a batch of 150 fattening pigs from 33 to 100 kg. A protracted growth period means production time would increase by two to three percent [20], reducing the number of pigs raised within the production year. At the national level, the disease burden of H1N1pdm09 is proportional to the portion of pig farmers experiencing increased production costs and reduced profit margins.

By using the first two years (2009–2010) of Norway’s active serosurveillance data of H1N1pdm09 in pig herds (unit of analysis), this paper examines the efficacy of using the compartmental disease model, DTMC, with a one-year interval as the time step in forecasting the herd prevalence of H1N1pdm09 in the Norwegian pig population.

## 2. Materials and Methods

### 2.1. Norwegian Pig Population H1N1pdm09 Active Serosurveillance

The FSA randomly tested ~500 pig herds yearly. Herds included all breeding herds (nucleus and multiplier) and sow pool herds [21,22] with ~10 pigs per herd test. For conventional sow herds, blood sampling from sows and boars took place at the slaughterhouse. In addition, ~50 random fattening pig herds each provided 10 blood samples at the slaughterhouse [16].

### 2.2. Laboratory Analyses and Herd Diagnoses

According to the standards of the World Organisation for Animal Health (OIE), laboratory serological diagnosis of H1N1pdm09 at the Norwegian Veterinary Institute in Oslo was a two-step process of initial ELISA for IAV antibodies followed by the haemagglutination test to identify the influenza strain [15,23]. So far, haemagglutination tests have consistently confirmed H1N1pdm09 as the only IAV infection in Norwegian pigs from 2009 to 2020 [24,25].

### 2.3. Surveillance Data 2009 to 2020 and Parameter Selection for DTMC Model

**Figure 1:**
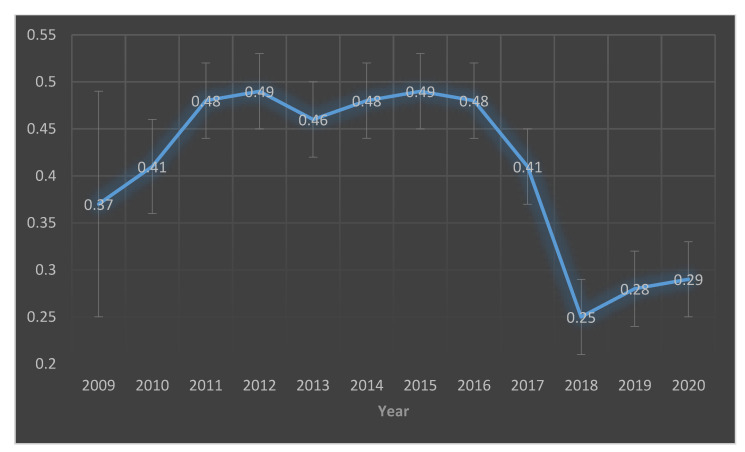
A longitudinal line plot of yearly prevalence of influenza A(H1N1)pdm09 virus infection in Norwegian pig herds obtained from the national active seroprevalence program 2009–2020. The 12% margin of error for year 2009 was much greater than the 4% for the other years because the disease outbreak occurred on 30 September 2009 leaving a smaller number (*n* = 54) of pig herds that were tested for the infection in 2009. A normal full year of sampling would involve ~500 pig herds.

In Figure 1, the longitudinal line graph shows that the herd prevalence escalated from 37% in 2009 to 48% in three years before plateauing and hovering at 48% until 2016 before sharply declining. Based on the initial trajectory from 2009 and 2016, the plan was to fit a DTMC model based on data from the first two years of surveillance from 2009 to 2010.

The prevalence for H1N1pdm09 is a binomial distribution of positive herds and negative herds with parameters *n* and *p*. It is a discrete probability distribution of the number of independent herd tests (*n*) with probability *p* for positive herd or *q* = 1 − *p* for uninfected [26].

I define a state *X* (national prevalence) as the probability that a pig herd in the population would test positive. Therefore, in this study, DTMC is a stochastic model describing a sequence of predicted herd prevalence in Norway from 2010 to 2020.

#### 2.3.1. Initial Herd Prevalence in 2009 (State *X*_0_)

The result of each herd test is dichotomous, i.e., positive or negative, like having a Bernoulli trial for each herd test. Bayesian inference after 54 herd tests or Bernoulli trials from 30 September 2009 (outbreak date) to 31 December 2009 gave the initial state *X*_0_ = 37% (95% CI 24–51%). In running the DTMC, this initial *X*_0_ (first row of Table 1) was the initial state vector, a 1 × 2 matrix [p,q] where *p* = 0.37 and *q* = 0.63 and *p* is the initial prevalence.

#### 2.3.2. Four Transition Probabilities for the Transition Matrix

**Figure 2:**
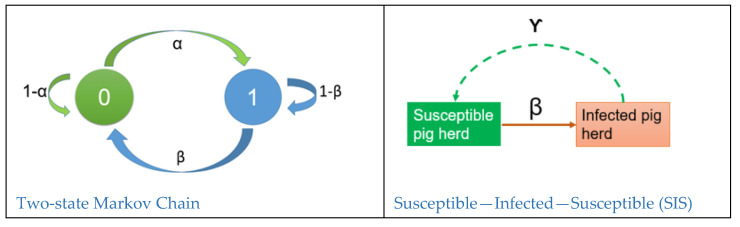
On the left, the diagram shows transition states of Norwegian pig herds in a two-state discrete-time Markov chain since 2009. In the first state (2009), a pig herd either existed as either uninfected (0) or infected (1). In the next time state, the uninfected herd could shift to the new state of infected with a probability *α* or remain in its uninfected status quo with a probability of 1 − *α*. Similarly, the infected herd could recover to an uninfected state in the next time step with a probability of *β* or remain infected with a probability of 1 − *β*. The transition probabilities satisfy the condition 0<α, β<1 Quite similar to the Markov model is another compartmental model, the SIS model. Parameters of the SIS model are infection rate (*β*) and recovery rate (ϒ).

Since the H1N1pdm09 incursion in 2009, each pig herd could exist in dichotomous states, i.e., either infected (1) or uninfected (0). In the next time step (a year later), the Two-state Markov Chain on the left in Figure 2 shows four possible transitions for each pig herd, which depend only on its current state, namely, an uninfected herd can either become infected (0_1) with a probability of *α*, or remain uninfected (0_0) with a probability of 1 − *α*. An infected herd, in the next time step, can recover to an uninfected state (1_0) with a probability of *β* or stay infected (1_1) with a probability of 1 − *β*.

#### 2.3.3. Equations for Discrete-Time Markov Chain Binomial Model

Let $\left\{Z_{i},i=1,2,\ldots \right\}$ be a stationary two-state Markov chain [27,28] with states 0 (uninfected) and 1 (infected), an initial vector or probability distribution (prevalence in 2009) denoted [p, q], in which $p=P\left(Z_{1}=1\right)\;$ and $q=P\left(Z_{1}=0\right)=1-p$, and transition probabilities $p_{i,j}=P\left(Z_{m+1}=j|Z_{m}=i\right)$ for *i, j* = 0, 1 and {m=1,2,…,10}.

The transition probabilities satisfy 0<α, β<1 and the transition matrix for DTMC has the following form:

$$P=\;\left[\begin{array}{cc} p_{00} & p_{01}\\ p_{10} & p_{11} \end{array}\right]=\left[\begin{array}{cc} 1-\alpha & \alpha \\ \beta & 1-\beta \end{array}\right]$$

$$\left[\begin{array}{cc} p_{m-1}, & q_{m-1} \end{array}\right]\times {\left[\begin{array}{cc} 1-\alpha & \alpha \\ \beta & 1-\beta \end{array}\right]}^{m}=\left[\begin{array}{cc} p_{m}, & q_{m} \end{array}\right]$$

$${\left[\begin{array}{cc} 0.37 & 0.63 \end{array}\right]}_{2009}\times {\left[\begin{array}{cc} 0.68 & 0.32\\ 0.34 & 0.66 \end{array}\right]}^{1}={\left[\begin{array}{cc} 0.44 & 0.56 \end{array}\right]}_{2010}$$

$$X_{1}=P\times X_{0}$$

$$X_{2}=P\times X_{1}$$

$$X_{3}=P\times X_{2}$$

where *X_i_* is the DTMCP and *i* = 2009, 2010,…, 2020 and *m* = *time* steps of transitions, where *m* = 1, 2, 3,…, 11; *m* = 1 is the transition step of 2009/2010, and *m* = 11 is the last transition step from 2019/2020.

## 3. Results

Retrospective examination of the national active surveillance data H1N1pdm09 in Norway 2009–2020 (Table 2) showed that the changing observed herd prevalence was commensurate with the varying observed transition probabilities for each year.

**Figure 3:**
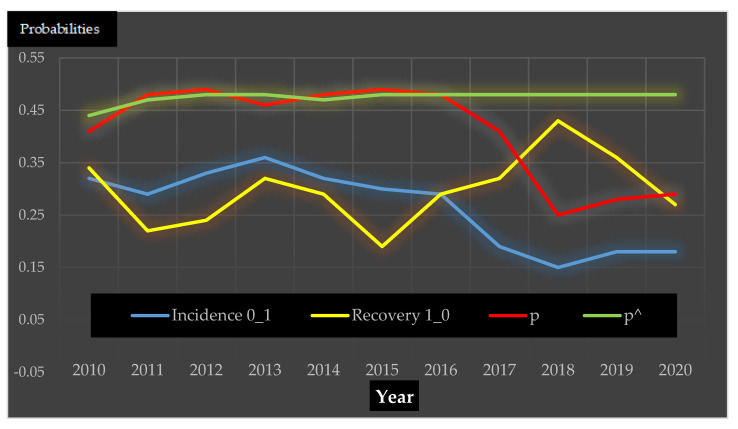
A comparative longitudinal line graph from 2009 to 2020 on four variables represented by the 4 coloured lines. 1. Red line—observed herd surveillance in pigs. 2. Green line—discrete-time Markov chain of predicted prevalence with 2009 herd prevalence as the first vector state (Table 1 and Table 2) and probability transition matrix based on incidence rate and recovery rate for the first two years of 2009 and 2010 (Table 2). 3. Blue line—herd incidence rate. 4. Yellow line—herd recovery rate. The latter two longitudinal measurements were possible with pig herds tested contiguously for two consecutive years.

Table 2 and Figure 3 show congruence (Pearson correlatio*n* = 0.88) between the DTMC projected prevalence (*p*^) and the observed surveillance prevalence (*p*), for the first seven years between 2010 and 2016. The *p*^ values were within the 95% confidence intervals of p shown in Table 2. After three time steps, the DTMC sequence *p*^ began converging in 2012 to a stationary state of 48% and remained statistically similar to *p* until 2016. After which, *p* diverged steeply downwards by 7% in 2017 and continued declining to 25% in 2018 before rising to 28% and 29% in 2019 and 2020, respectively. Table 2 shows the decline was due to a sharp 30% drop in new infections (0_1) and a 10% increase in recovery rate (1_0).

## 4. Discussion

Based on 11 years of observed data, this retrospective study demonstrated that the construction of a DTMC model based on the first two years (2009–2010) of surveillance could project H1N1pdm09 prevalence for the first seven years (2010–2016) with good congruence with the observed data. The predictive value was however, limited after 2016 because while the predicted prevalence continued unchanged after converging in 2012, the observed prevalence diverged by declining sharply. The convergence of the DTMC generated prevalence is a trait of the fixed probability transition matrix (incidence and recovery) achieved by longitudinally generating incremental *p^* values until convergence. The DTMC in this study achieved convergence to a stationary state of 48% after three time steps. Validating with the actual surveillance data retrospectively, the DTMCP or *p^* showed high correlation (Pearson correlatio*n* = 0.88) with the *p* for the only first seven years (Table 2 and Figure 3). Congruence ended after 2016 with the observed prevalence declining markedly to 41% (2017) and 25% (2018) before reversing the decline by climbing to 28% (2019) and 29% (2020). 

Climbing quickly to 48% by 2012 (after 3 years) the herd prevalence remained stable for 5 years, indicating that disease dynamics of pig herds in terms of herd infectious rates and herd recovery rates were in equilibrium. This equilibrium clearly ended after 2016 because the force of infection for pig herds in the population had changed. One can see in Figure 3, the divergence of increasing herd recovery rates (yellow line) and the declining herd incidence (blue line). To understand this sudden divergence after 2016, the Human influenza active surveillance data from the Norwegian Institute of Public health revealed the declining H1N1pdm09 prevalence in pigs parallel that with the changing IAV strain dominance in human influenza seasons. Just as the infected human population had introduced H1N1pdm09 to Norwegian pigs in 2009 [4,18], persistent human H1N1pdm09 infections in Norway could hypothetically have maintained the force of pig herd infections. Since 2009, H1N1pdm09 remained the dominant human IAV strain until the 2015/2016 influenza season with a dominance of 91% before declining steeply by nearly a 100% to only 1% in the next winter influenza (20016/2017). The dominance of IAV H3N2 strain was 99% [29,30]. In just one year, the human contribution of H1N1pdm09 to the force of pig herd infection had abruptly disappeared.

Serosurveillance detects antibodies, and so one may argue that positive herds were not necessarily new infections from active virus, especially in pig herds that tested positive contiguously for consecutive years. However, if one considers the rapid turnover of sows in pig production in Norway, where sows on average do not stay longer than 2 years, a sow herd that tested positive more than 2 years apart was a new infection. Sustained detection of antibodies in the same pig herd indicates that there is continual new infection of susceptible pigs replenished continuously to commercial sow herds (50% of pig herds in Norway). Generally, the majority of sow herds in Norway do not practise all-in–all-out husbandry [17]. 

Although both DTMC and SIS models share probabilistic parameters of incidence and recovery rates, the SIS model is not ideal for pig herd infections. The infectious disease dynamics for H1N1pdm09 in pig herds do not satisfy the basic assumptions of the classic SIR model having homogenous mixing of the infected and susceptible populations and that the total population is constant in time. The pig population is never constant because of the high turnover rate of pigs. For example, fattening pigs go to the slaughterhouse at 6 months of age. There is also no homogenous mixing of pig herds since they are geographically stationary at their farm locations thus ruling out herd-to-herd transmissions. Rather, the force of pig herd infection comes more likely from a complex web of human-driven husbandry activities since humans have been the most likely vector and reservoir as long as H1N1pdm09 was the dominant IAV strain during the human flu seasons. Therefore, from a broad perspective and by dispensing with specific disease transmission conditions, DTMC, needed only the first two years of probability data for parameters, is the preferred model in this study. Simplistic but efficacious at least for the first seven years from outbreak, a period long enough for animal health authorities to estimate the height (converged prevalence) of the disease burden and evaluate it with animal health economics.

Yet simplicity in the DTMC model may not always be advantageous because it is an aggregated representation of all types in pig production. It does not account for differential infectious dynamics related to different types of pig production. Moreover, a recent study in Norway showed that fattening pig herds tend to have lower incidence rates and hence lower prevalence compared to conventional sow herds [17]. Refinement of the model to reflect production types will have greater expediency for animal health economics in targeting a certain production type, e.g., fattening herds as opposed to sow herds.

## 5. Conclusions

Even though some would consider the DTMC an oversimplified model in portraying the infectious dynamics of influenza A(H1N1)pdm09 in pig herds, animal health authorities can nevertheless use it to predict disease burden during the early phase of an exotic disease incursion. The predicted convergence was also accurate in measuring the highest point of the observed herd prevalence, which is a useful yardstick for animal health economics evaluation. As the H1N1pdm09 was widely prevalent in both the human and pig populations, the interrelated disease dynamics is an important consideration for using the DTMC in forecasting disease prevalence. Hence, it is imperative that the estimation of national disease burden must take a One Health approach for a holistic socioeconomic evaluation of intervention measures.

## Figures and Tables

**Table 1:** Active serosurveillance gave yearly prevalence estimates of influenza A(HIN1)pdm09 infection in pig herds. Testing every year from 2009 to 2020 involved 500–700 randomly selected pig herds, a coverage of between one-quarter to one-third of total pig herds in Norway.

Year	Total Pig Herds	Herds Tested	Population Proportion	Herds Positive	Prevalence	95% CI
2009	2546	452 (54 *)	2%	20	0.37	(25–52)
2010	2441	459	19%	189	0.41	(37–46)
2011	2346	730	31%	353	0.48	(45–52)
2012	2213	764	35%	378	0.49	(46–53)
2013	2178	737	34%	338	0.46	(42–50)
2014	2117	622	29%	296	0.48	(44–52)
2015	2141	568	27%	280	0.49	(45–53)
2016	2180	564	26%	271	0.48	(44–52)
2017	1955	548	28%	225	0.41	(37–45)
2018	2038	533	26%	134	0.25	(22–29)
2019	1853	545	29%	153	0.28	(24–32)
2020		535		154	0.29	(25–33)

* 30 September 2009 was the date of outbreak detection of influenza A(H1N1)pdm09 virus infection in Norwegian pigs. Sampling frame in 2009 and prevalence calculations were valid only after that date.

**Table 2:** Observed transition probabilities in surveillance data based on the incidence rates and recovery rates of pig herds that had consecutive years testing. The four transition probabilities were (a) negative herd remaining negative the following year (0_0), (b) negative herd turning positive the following year (0_1), (c) positive herd remaining positive following year (1_1) and (d) positive herd turning negative the following year (1_0). For the discrete-time Markov chain (DTMC) iterations, only the initial incidence rates and recovery rates derived from the 2009 to 2010 data became the fixed transition probability matrix. The last three columns of the table compare the observed prevalence (*p*) with the corresponding DTMC of projected herd prevalence (*p^*). To measure the congruence between the two prevalence, the last column (*p^ − p*) shows the difference between observed *p* and *p^*.

		Transition Probabilities (*n* = Pig Herds)				DTMC Projected Prevalence	Surveillance Prevalence	DTMC— Observed
Year	No. of Pig Herds	0_0	Incidence 0_1	1_1	Recovery 1_0	*p*^	*p* (95 CI)	*p*^ − *p*
2009	0	0	0	0	0	37%	37% (25–52)	0%
2010	113	0.68	0.32	0.66	0.34	44%	41% (37–46)	3%
2011	241	0.71	0.29	0.78	0.22	47%	48% (45–52)	−1%
2012	447	0.67	0.33	0.76	0.24	48%	49% (46–53)	−1%
2013	461	0.64	0.36	0.68	0.32	48%	46% (42–50)	2%
2014	433	0.68	0.32	0.71	0.29	47%	48% (44–52)	−1%
2015	348	0.7	0.3	0.81	0.19	48%	49% (45–53)	−1%
2016	348	0.71	0.29	0.71	0.29	48%	48% (44–52)	0%
2017	332	0.81	0.19	0.68	0.32	48%	41% (37–45)	7%
2018	237	0.85	0.15	0.57	0.43	48%	25% (22–29)	23%
2019	315	0.82	0.18	0.64	0.36	48%	28% (24–32)	20%
2020	188	0.82	0.18	0.73	0.27	48%	29% (25–33)	19%

## Data Availability

All active influenza surveillance data are available on the public website of the Norwegian Veterinary institute–www.vetinst.no (accessed on 15 July 2021). Human active influenza surveillance data are available on the website of the Norwegian institute of public health’s website-www.fhi.no (accessed on 15 July 2021).
